# Linc-DYNC2H1-4 promotes EMT and CSC phenotypes by acting as a sponge of miR-145 in pancreatic cancer cells

**DOI:** 10.1038/cddis.2017.311

**Published:** 2017-07-13

**Authors:** Yuran Gao, Zhicheng Zhang, Kai Li, Liying Gong, Qingzhu Yang, Xuemei Huang, Chengcheng Hong, Mingfeng Ding, Huanjie Yang

**Affiliations:** 1 School of Life Science and Technology, Harbin Institute of Technology, Harbin, China; 2 Department of General Surgery, Fourth Affiliated Hospital of Harbin Medical University, Harbin, China

## Abstract

The acquisition of epithelial–mesenchymal transition (EMT) and/or existence of a sub-population of cancer stem-like cells (CSC) are associated with malignant behavior and chemoresistance. To identify which factor could promote EMT and CSC formation and uncover the mechanistic role of such factor is important for novel and targeted therapies. In the present study, we found that the long intergenic non-coding RNA linc-DYNC2H1-4 was upregulated in pancreatic cancer cell line BxPC-3-Gem with acquired gemcitabine resistance. Knockdown of linc-DYNC2H1-4 decreased the invasive behavior of BxPC-3-Gem cells while ectopic expression of linc-DYNC2H1-4 promoted the acquisition of EMT and stemness of the parental sensitive cells. Linc-DYNC2H1-4 upregulated ZEB1, the EMT key player, which led to upregulation and downregulation of its targets vimentin and E-cadherin respectively, as well as enhanced the expressions of CSC makers Lin28, Nanog, Sox2 and Oct4. Linc-DYNC2H1-4 is mainly located in the cytosol. Mechanically, it could sponge miR-145 that targets *ZEB1*, *Lin28*, *Nanog*, *Sox2*, *Oct4* to restore these EMT and CSC-associated genes expressions. We proved that *MMP3*, the nearby gene of linc-DYNC2H1-4 in the sense strand, was also a target of miR-145. Downregulation of *MMP3* by miR-145 was reverted by linc-DYNC2H1-4, indicating that competing with miR-145 is one of the mechanisms for linc-DYNC2H1-4 to regulate *MMP3*. In summary, our results explore the important role of linc-DYNC2H1-4 in the acquisition of EMT and CSC, and the impact it has on gemcitabine resistance in pancreatic cancer cells.

Pancreatic ductal adenocarcinoma (PDAC) is one of the most commonly diagnosed cancers and the fourth leading cause of cancer-related death.^1^ Gemcitabine (2′,2′-difluorodeoxycytidine), a deoxycytidine analog, represents the first line intervention for the treatment of advanced PDAC, and demonstrates prolonged overall survival time and improved life quality.^2, 3, 4^ However, resistance of cancer cells to gemcitabine, either intrinsic or acquired during treatment, has been frequently observed in patients and is considered as the major reason for cancer progression.^5^ Emerging evidence suggests the association between PDAC chemoresistance and the acquisition of epithelial–mesenchymal transition (EMT) phenotype and/or the existence of a sub-population of cancer stem-like cells (CSC) within the tumor mass.^6, 7^ These chemoresistant cancer cells are refractory to gemcitabine and highly prone to metastasize.^6^

Recently, long non-coding RNAs (lncRNAs), RNA molecules with >200 nt in length, have been reported involved in the regulation of CSC and EMT phenotypes in PDAC. Metastasis associated lung adenocarcinoma transcript 1 (MALAT1), which was originally discovered in association with metastatic behavior of non small cell lung cancer, has been reported to promote metastasis in PDAC.^8, 9^ LncRNA ROR (Regulator of Reprogramming) was found to upregulate CSC maker Nanog or EMT inducer ZEB1, leading to increased pancreatic cancer invasion and tumorigenesis.^10, 11^ LncRNA H19, an imprinted gene was also found to promote PDAC cell invasion and migration by increasing HMGA2-mediated EMT through antagonizing let-7.^12^

LncRNAs are transcribed from the intergenic regions, overlapping (in sense or antisense orientation), or intronic to protein-coding genes, among which long intergenic non-coding RNAs (lincRNAs) account for 50% and represent one of the most mystery groups with functions needed to be defined.^13, 14, 15^ In the present study, we found that the linc-DYNC2H1-4 was upregulated in BxPC-3-Gem cell line with acquired gemcitabine resistance. Downregulation of linc-DYNC2H1-4 was associated with decreased invasive behavior of BxPC-3-Gem cells while overexpression of linc-DYNC2H1-4 promoted the acquisition of CSC and EMT phenotypes of parental gemcitabine-sensitive BxPC-3 cells. Mechanically, linc-DYNC2H1-4 competed with miR-145, leading to upregulation of its targets Lin28, Nanog, Sox2, Oct4, ZEB1 and MMP3 that are involved in EMT and CSC regulation.

## Results

### Gemcitabine-resistant pancreatic cancer cells exert enhanced EMT and CSC properties

**Figure 1:**
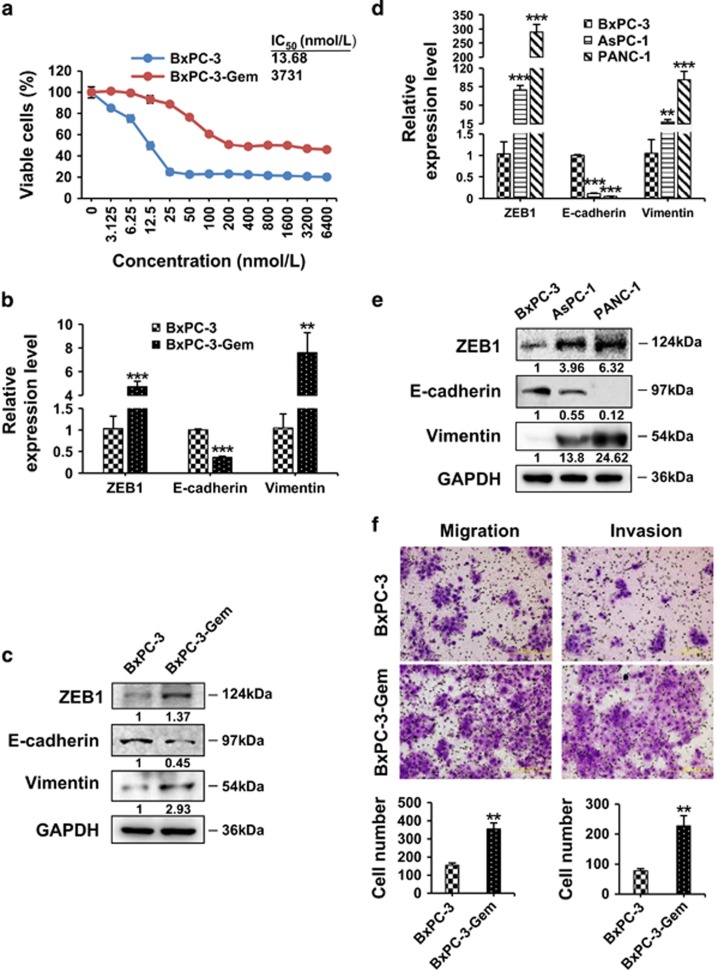
Gemcitabine-resistant pancreatic cancer cells exhibit enhanced EMT potential. (**a**) MTT assay after 72 h treatment with gemcitabine in BxPC-3-Gem and parental BxPC-3 cells. (**b**–**e**) The expression levels of EMT markers, ZEB1, E-cadherin and vimentin were detected by RT-qPCR (**b**,**d**) and western blotting (**c** and **e**) in BxPC-3, BxPC-3-Gem, AsPC-1 and PANC-1 cells. GAPDH was used as a loading control (**c** and **e**). Bands intensities normalized to GAPDH were shown (**f**) The migration and invasion abilities of BxPC-3 and BxPC-3-Gem cells were measured by Transwell assay (Scale bar, 200 μm). The data shown were from three independent experiments. **P<0.01; ***P<0.001 *versus* BxPC-3

We established a gemcitabine-resistant cell line (BxPC-3-Gem) through exposing parental BxPC-3 cells to increased concentrations of gemcitabine for 16 months. BxPC-3-Gem showed ~270-fold enhanced resistance to gemcitabine compared with BxPC-3 cells as reflected by IC_50_ (Figure 1a). As only a small population with EMT and/or CSC phenotypes remained after each selection, we compared the EMT/CSC properties between BxPC-3-Gem cells and BxPC-3. ZEB1, which initiates the EMT program through downregulation of E-cadherin and upregulation of vimentin,^16^ was increased in BxPC-3-Gem cells, along with decrease of E-cadherin and increase of vimentin (Figures 1b and c). High expression of ZEB1 and vimentin, and low exprssion of E-cadherin, were verified in pancreatic cancer cell lines with intrinsic gemcitabine resistance, AsPC-1 and PANC-1 (Figures 1d and e). As EMT contributes to metastatic behavior of cancer cells,^6, 7^ we determined the cell motility in the two lines through Transwell assay. Both invasion and migration abilities were significantly increased in BxPC-3-Gem cells compared with BxPC-3 (Figure 1f).

**Figure 2:**
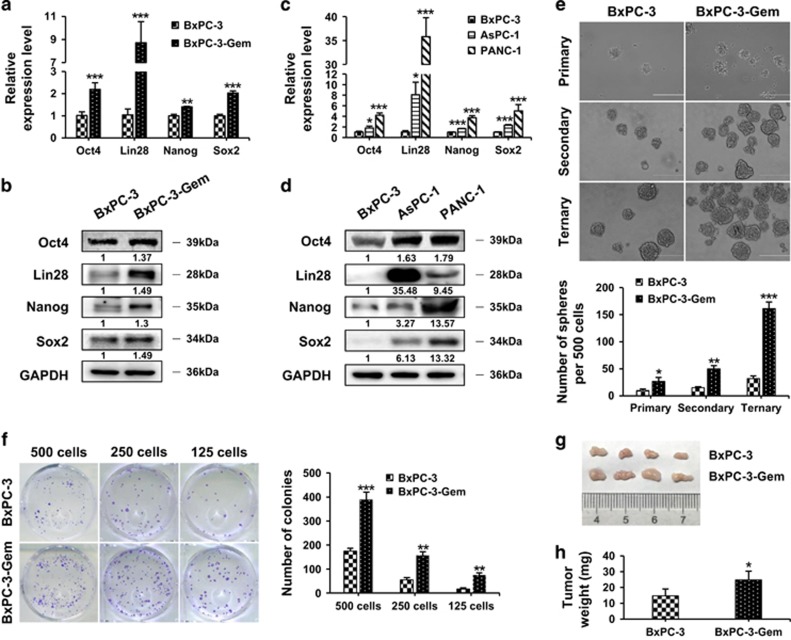
Gemcitabine-resistant pancreatic cancer cells exert enhancedcancer stem cell characteristics. (**a**–**d**) The expression levels of CSC markers, Oct4, Lin28, Nanog and Sox2 were determined by RT-qPCR (**a**,**c**) and western blotting (**b**,**d**) in BxPC-3, BxPC-3-Gem, AsPC-1 and PANC-1 cells. The data shown were from three independent experiments. Bands intensities normalized to GAPDH were shown. (**e**) Pancreatosphere formation. Scale bar, 200 μm. (**f**) Colony formation. (**g**) Tumorigenecity *in vivo* (*n*=4). (**h**) Tumor weights were measured. *P<0.05; **P<0.01; ***P<0.001 *versus* BxPC-3

BxPC-3-Gem also showed increased CSC properties compared with parental BxPC-3 cells. Lin28, the CSC marker, was highly induced at mRNA and protein levels in BxPC-3-Gem compared with BxPC-3 cells (Figures 2a and b). The other three CSC markers Oct4, Nanog and Sox2, were also significantly highly expressed in BxPC-3-Gem cells (Figures 2a and b). Compared with BxPC-3, higher expression levels of these CSC makers were also detected in gemcitabine-resistant AsPC-1 and PANC-1 cells, among which Lin28 exerted remarkable overexpression (Figures 2c and d).

Self-renewal is a key property of cancer stem cells, which can be determined by serial sphere formation. Sphere-forming ability was evaluated for three generations for BxPC-3-Gem and parental cells. The numbers of primary as well as secondary and ternary pancreatospheres formed by BxPC-3-Gem were all significantly increased compared with those formed by parental cells (Figure 2e), indicating the enhanced *in vitro* self-renewal capability of BxPC-3-Gem cells. BxPC-3-Gem also showed greater abilities to form colonies compared with BxPC-3 cells evaluated by limit dilution colony formation assay. With cell numbers dilutions (500 to 250, and further to 125) the ratios of colony numbers between BxPC-3-Gem and BxPC-3 cells were increased (2.2, 2.8 and 4.4-fold, respectively), showing more significant difference in colony formation when dilution rate increased (Figure 2f).

Tumorigenicity *in vivo* was used to evaluate the existence of CSCs. BxPC-3 or BxPC-3-Gem cells were injected subcutaneously into nude mice at different numbers (10^3^, 10^5^ and 10^7^ per inoculation). Both cells failed to form tumors at lower numbers (10^3^ and 10^5^ per inoculation, data not shown), but developed tumors with inoculation of 10^7^ cells (Figure 2g), and increased tumorigenicity was observed for BxPC-3-Gem compared with BxPC-3 cells as shown by increased tumor weight (Figure 2h). In another experiment, gemcitabine-resistant PANC-1 cells formed tumors at 10^6^ per inoculation (4/4), whereas the sensitive BxPC-3 cells failed to form tumors at the same number, but developed tumors at 10^7^ per inoculation (4/4) (Table 1). These results show that gemcitabine-resistant cells have greater tumorigenicity compared with gemcitabine-sensitive pancreatic cancer cells.

### Upregulation of linc-DYNC2H1-4 in gemcitabine-resistant pancreatic cancer cells

**Figure 3:**
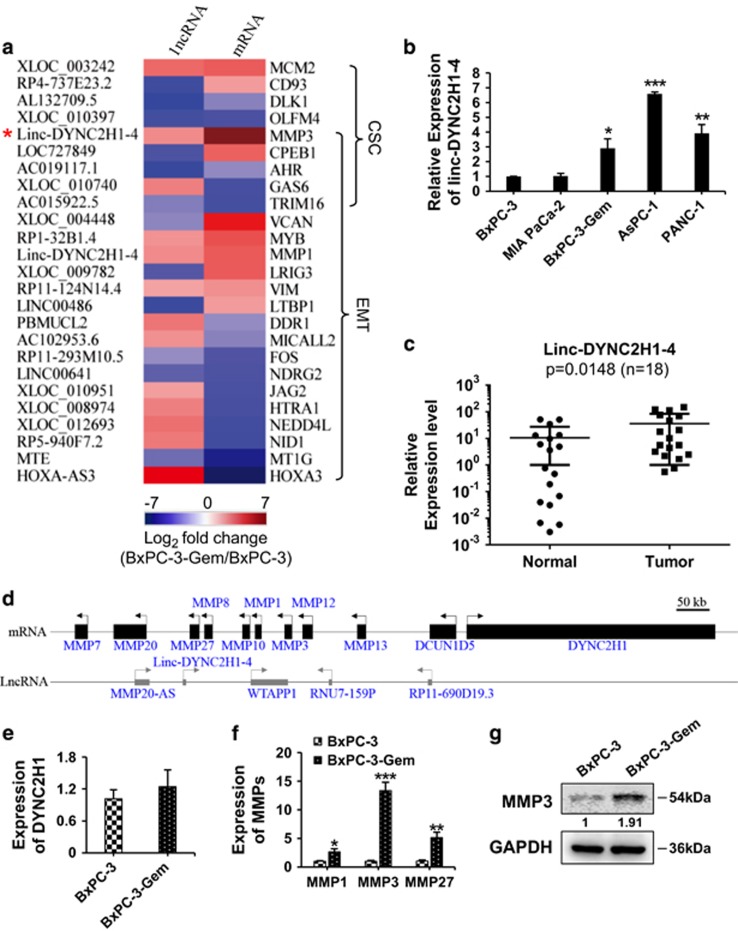
Linc-DYNC2H1-4 and MMP3 are upregulated in gemcitabine-resistant pancreatic cancer cells. (**a**) The log2 fold change of lncRNAs and their nearby coding genes that associated with CSC and EMT was presented by heat map. (**b**,**c**) Expression of linc-DYNC2H1-4 in pancreatic cancer cell lines (**b**), PDAC and paired adjacent pancreatic tissues (**c**) were determined by RT-qPCR. (**d**) The locus map of linc-DYNC2H1-4 and nearby genes (black), as well as lncRNAs (gray). (**e**–**g**) The expression levels of linc-DYNC2H1-4 and nearby genes: *DYNC2H1*, *MMP1*, *MMP3* and *MMP27* were determined by RT-qPCR (**e**,**f**) and western blotting (**g**) in BxPC-3 and BxPC-3-Gem cells. The data shown were from three independent experiments. *P<0.05; **P<0.01; ***P<0.001 *versus* BxPC-3

To explore the underlying mechanisms responsible for the enhanced EMT and CSC properties in gemcitabine-resistant cells, we performed lncRNA and mRNA array analysis. Downregulated and upregulated genes with over twofold changes in BxPC-3-Gem compared with BxPC-3 were displayed in Figure 3a, among which linc-DYNC2H1-4 was chosen as its nearby gene *MMP3* was involved in both EMT and CSC regulation (Figure 3a). RT-qPCR confirmed that linc-DYNC2H1-4 was overexpressed in BxPC-3-Gem as well as other gemcitabine-resistant cells compared with gemcitabine-sensitive BxPC-3 and MIA PaCa-2 cells (Figure 3b). Higher expression levels of linc-DYNC2H1-4 were detected in PDAC in comparison with adjacent normal tissues (Figure 3c). The closest gene to linc-DYNC2H1-4 in the sense strand is *DYNC2H1*, which it was named after (Figure 3d). No significant difference of *DYNC2H1* expression was found between BxPC-3-Gem and BxPC-3 (Figure 3e). In contrast, the expressions of nearby genes in the antisense strand, *MMP1*, *MMP3* and *MMP27*, were significantly different, among which *MMP3* showed the most significant difference (Figure 3f). MMP3 protein was also upregulated in BxPC-3-Gem compared with BxPC-3 cells (Figure 3g).

### Knockdown of linc-DYNC2H1-4 suppresses EMT and CSC properties in gemcitabine-resistant pancreatic cancer cells

**Figure 4:**
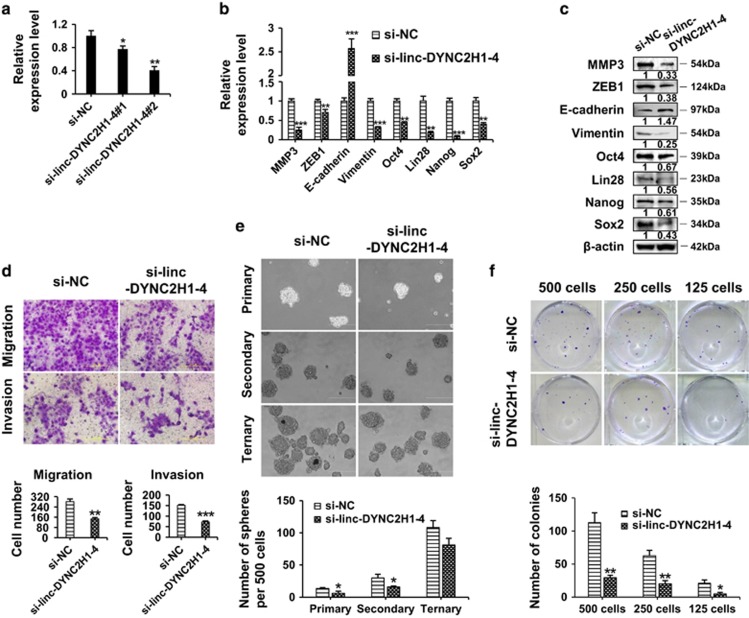
Knockdown of linc-DYNC2H1-4 suppresses EMT and CSC properties in gemcitabine-resistant pancreatic cancer cells. BxPC-3-Gem cells were transfected with siRNA for linc-DYNC2H1-4 (si-linc-DYNC2H1-4) or negative control (si-NC) for 48 h. (**a**) The silencing efficiency was analyzed by RT-qPCR. (**b** and **c**) The mRNA and protein expression levels of EMT- and CSC-associated genes were analyzed by RT-qPCR (**b**) and western blotting (**c**), respectively. (**d**–**f**) The migration and invasion abilities, self-renewal capacity and colony-forming ability of BxPC-3-Gem cells were measured by Transwell assay (**d**), three generations of sphere formation assay (**e**) and limited dilution colony-forming assay (**f**), respectively. The data shown were from three independent experiments. *P<0.05; **P<0.01; ***P<0.001 *versus* control group. Scale bar, 200 *μ*m

To address the role of linc-DYNC2H1-4 in the formation of EMT and CSC phenotypes in gemcitabine-resistant cells, we transfected BxPC-3-Gem cells with siRNAs targeting linc-DYNC2H1-4. Both siRNAs significantly decreased the expressions of linc-DYNC2H1-4 (Figure 4a). As siRNA#2 showed better silencing effect than siRNA#1, it was used in the further study. After transfection with linc-DYNC2H1-4 siRNA, the levels of MMP3, ZEB1 and vimentin, as well as Oct4, Lin28, Nanog and Sox2 were significantly decreased, while the level of E-cadherin was increased (Figures 4b and c). Relative to these molecular alterations, knockdown of linc-DYNC2H1-4 inhibited the EMT properties of BxPC-3-Gem cells, as shown by ~twofold decreased cell numbers of migration and invasion compared with control (Figure 4d). Knockdown of linc-DYNC2H1-4 also led to ~twofold drop of primary and secondary pancreatospheres compared with control (Figure 4e). However, the difference between the two groups in secondary pancreatospheres was less than that in the primary pancreatospheres (2.2 *versus* 1.9), and no significant difference was observed for ternary pancreatospheres formation between the two groups (Figure 4e). Knockdown of linc-DYNC2H1-4 inhibited the colony formation ability of BxPC-3-Gem cells as shown in limit dilution colony formation assay. The fewer cells seeded, the more difference in colony formation was observed between knockdown and control groups (Figure 4f).

### Overexpression of linc-DYNC2H1-4 promotes EMT and CSC phenotypes in gemcitabine-sensitive pancreatic cancer cells

**Figure 5:**
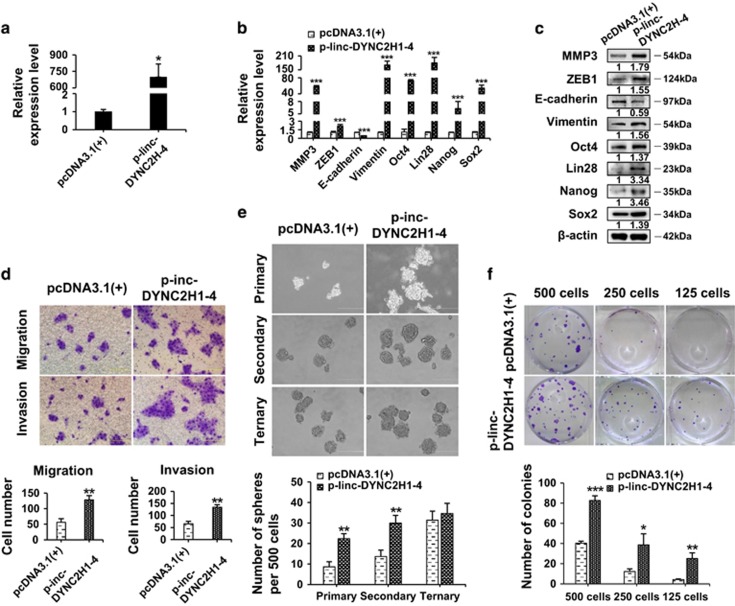
Overexpression of linc-DYNC2H1-4 promotes EMT and CSC properties in gemcitabine-sensitive pancreatic cancer cells. (**a**–**c**) BxPC-3 cell were transfected with linc-DYNC2H1-4 expression vector (p-linc-DYNC2H1-4) or pcDNA3.1(+) empty vector for 24 h, the expressions of linc-DYNC2H1-4 (**a**) as well as EMT- and CSC-associated genes were analyzed by RT-qPCR (**b**) and western blotting (**c**). (**d**–**f**) The migration and invasion abilities, self-renewal capacity and colony-forming ability of BxPC-3 cells after transfection were measured by Transwell assay (**d**), three generations of tumor sphere formation assay (**e**) and limited dilution colony-forming assay (**f**), respectively. The data shown were from three independent experiments. *P<0.05; **P<0.01; ***P<0.001 *versus* pcDNA3.1(+) group. Scale bar, 200 *μ*m

To determine whether linc-DYNC2H1-4 would promote the CSC and EMT phenotypes in gemcitabine-sensitive cells, it was overexpressed in BxPC-3 cells (Figure 5a). Overexpression of linc-DYNC2H1-4 caused significant upregulation of ZEB1, vimentin, Oct4, Lin28, Nanog, Sox2 and downregulation of E-cadherin at mRNA and protein levels (Figures 5b and c). Overexpression of linc-DYNC2H1-4 also promoted EMT and CSC phenotypes in gemcitabine-sensitive BxPC-3 cells as shown by increased migration and invasion (Figure 5d) as well as more than 2-fold increase of primary and secondary pancreatospheres (Figure 5e). However the difference of secondary pancreatospheres between linc-DYNC2H1-4 and control groups were less than that of the primary pancreatospheres (2.6 *versus* 2.2), and ternary pancreatospheres showed no significant difference between two groups (Figure 5e). These results were similar to knockdown manipulation, suggesting that effects of transient transfection would wear off in long time culture for the generation of ternary pancreatospheres. Overexpression of linc-DYNC2H1-4 promoted the colony formation ability of BxPC-3 cells. Double dilutions of BxPC-3 cells with overexpressed linc-DYNC2H1-4 formed equal or even more colonies than control cells seeded without dilution (Figure 5f).

### Linc-DYNC2H1-4 functions as a sponge of miR-145 in pancreatic cancer cells

**Figure 6:**
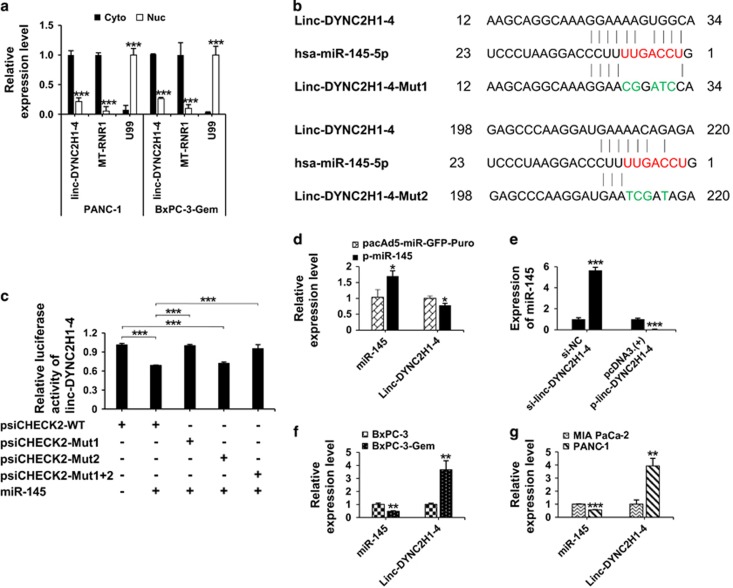
Linc-DYNC2H1-4 functions as a ceRNA of miR-145 in pancreatic cancer cells. (**a**) Intracellular location of linc-DYNC2H1-4. Linc-DYNC2H1-4 level in the nuclear (Nuc) and cytoplasmic (Cyto) fractions was determined by RT-qPCR in PANC-1 and BxPC-3-Gem cells. U99 and MT-RNR1 were used as nuclear and cytoplasmic marker, respectively. (**b**) Linc-DYNC2H1-4 was predicted containing two binding sites to miR-145. Seed sequences of miR-145 were shown in red. Linc-DYNC2H1-4 mutations at each binding site were indicated in green. (**c**) Luciferase reporter constructs containing wild-type binding site (psiCHECK2-WT), mutation at each site (psiCHECK2-Mut1, psiCHECK2-Mut2) or both (psiCHECK2-Mut1+2) were transfected in BxPC-3-Gem cells in the presence of p-miR-145 (+) or empty vector (−) for 24 h. Luciferase activity was determined. (**d–f**) RT-qPCR analysis. Linc-DYNC2H1-4 expression in BxPC-3-Gem cells after miR-145 overexpression (**d**). MiR-145 expression in BxPC-3-Gem after linc-DYNC2H1-4 knockdown or in BxPC-3 cells after linc-DYNC2H1-4 overexpression (**e**). Endogenous expression levels of miR-145 and linc-DYNC2H1-4 were compared in BxPC-3-Gem *versus* parental cells (**f**), and PANC-1 *versus* MIA PaCa-2 (**g**). *P<0.05; **P<0.01; ***P<0.001 *versus* empty vector group

Given that linc-DYNC2H1-4 can regulate multiple genes which are important for EMT and CSC properties, we speculated that it might work as a sponge to inhibit certain miRNAs so as to liberate their target mRNA transcripts. First, we characterized the intracellular location of linc-DYNC2H1-4. Nuclear and cytosolic fractions were separated from BaPC-3-Gem and PANC-1 cells. RT-qPCR revealed that linc-DYNC2H1-4 was mainly located in the cytosol (Figure 6a), supporting the possibility that linc-DYNC2H1-4 acts as a sponge of miRNAs. Considering known functions of miRNAs, miR-145 was selected for further study.^10, 17, 18, 19, 20^ Two binding sequences in the linc-DYNC2H1-4 transcripts were found pairing with miR-145 (Figure 6b). Luciferase reporters containing the linc-DYNC2H1-4 wild type (psiCHECK2-WT), or mutations at one (psiCHECK2-Mut1, psiCHECK2-Mut2) or both (psiCHECK2-Mut1+2) putative miR-145 binding sites were constructed. Transfection of miR-145 into BxPC3-Gem cells reduced the luciferase activity of the wild-type linc-DYNC2H1-4 reporter (Figure 6c). Mutation of the first binding site significantly reduced the effects of miR-145 on luciferase activity while mutation of the second binding site had no effect (Figure 6c), indicating that the first binding site seemed to be the interaction site. RT-qPCR analysis showed that miR-145 overexpression led to a marked decrease in linc-DYNC2H1-4 expression (Figure 6d). We then examined whether miR-145 level would be affected by linc-DYNC2H1-4. The expression of miR-145 was reduced by ~25-fold upon linc-DYNC2H1-4 overexpression in BxPC-3 cells, while it was increased by ~6-fold as linc-DYNC2H1-4 was knocked down in BxPC-3-Gem cells (Figure 6e). In addition, BxPC-3-Gem cells with higher expression of linc-DYNC2H1-4 had lower level of miR-145 compared with parental BxPC-3 cells (Figure 6f), showing endogenous expression levels of miR-145 and linc-DYNC2H1-4 were negatively correlated with each other. The negative correlation between miR-145 and linc-DYNC-2H1-4 was confirmed in MIA PaCa-2 and PANC-1 with differential gemcitabine resistance (Figure 6g; Supplementary Figure 1). In brief, these results demonstrate that miR-145 directly binds to linc-DYNC2H1-4 and that a reciprocal repression occurs between linc-DYNC2H1-4 and miR-145.

### miR-145 targets EMT and CSC markers which are upregulated by linc-DYNC2H1-4 in pancreatic cancer cells

**Figure 7:**
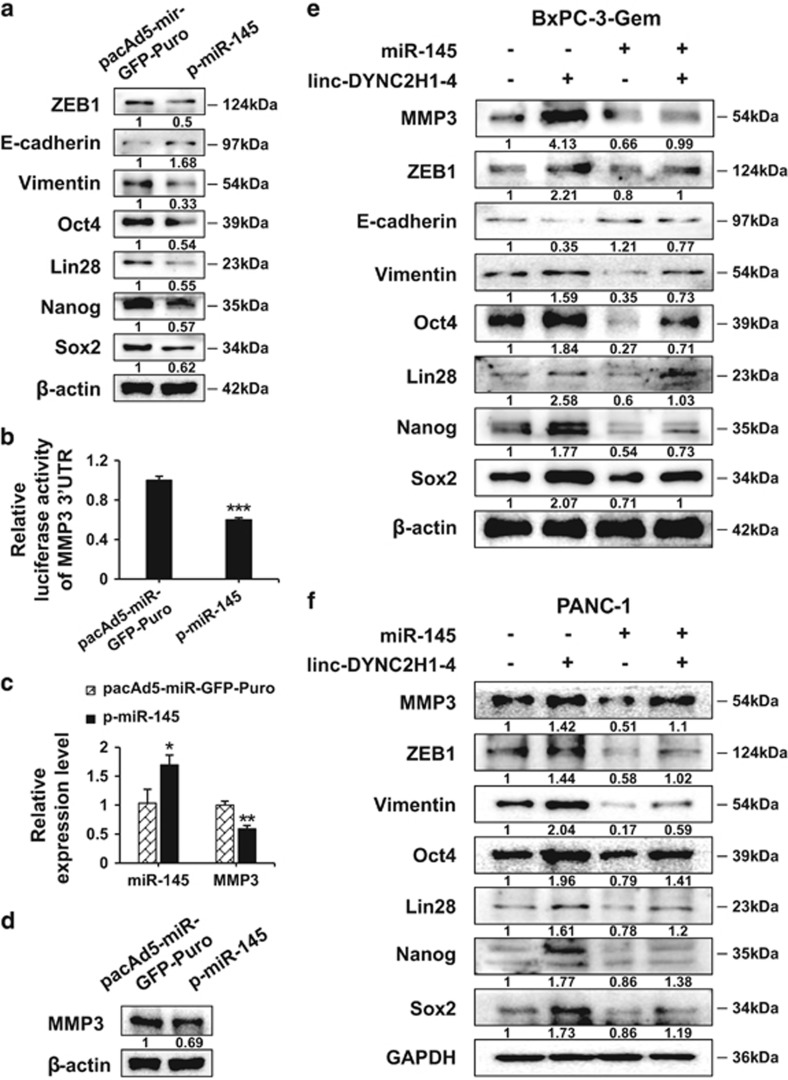
miR-145 targets EMT and CSC markers which are upregulated by linc-DYNC2H1-4. (**a**) BxPC-3-Gem cells were transfected with p-miR-145 overexpression vector or empty vector for 24 h, linc-DYNC2H1-4 upregulated genes, Oct4, Lin28, Nanog Sox2 and ZEB1 as well as its target genes E-cadherin and vimentin were detected by western blotting. (**b**–**d**) MiR-145 inhibits *MMP3* in pancreatic cancer cells. Luciferase reporter activity in BxPC-3-Gem cells co-transfected with miR-145 expression or empty vector together with a luciferase reporter vector containing the wild-type MMP3 3′ UTR (**b**). BxPC-3-Gem cells were transfected with empty or miR-145 overexpression vector for 24 h, expressions of miR-145 and MMP3 were analyzed by RT-qPCR (**c**) and western blotting (**d**). BxPC-3-Gem cells (**e**) and PANC-1 cells (**f**) were transfected with linc-DYNC2H1-4 overexpression vector (linc-DYNC2H1-4), or miR-145 expression vector (miR-145) alone or together for 24 h, the protein expression levels of EMT- and CSC-associated genes were detected by western blotting. The data shown were from three independent experiments. *P<0.05; **P<0.01 and ***P<0.001 *versus* control

MiR-145 could promote tumor progression by inhibiting Oct4, Lin28, Nanog, Sox2 and ZEB1 in different cancer models.^17, 18, 19, 20^ Overexpression of miR-145 resulted in downregulation of Oct4, Lin28, Nanog, Sox2 and ZEB1 in BxPC3-Gem cells (Figure 7a), confirming that miR-145 targets all these molecules in pancreatic cancer. As transcription factors, ZEB1/2 are able to initiate an EMT program through downregulation of E-cadherin and upregulation of vimentin.^16^ MiR-145 overexpression led to increase of E-cadherin and decrease of vimentin, in accordance with ZEB1 protein level (Figure 7a). Then, we were interested in whether miR-145 could regulate the nearby gene *MMP3* which was also upregulated by linc-DYNC2H1-4. Luciferase reporter gene assay showed that *MMP3* reporter activity dropped by ~twofold with miR-145 co-transfection (Figure 7b). Transfection of miR-145 led to ~twofold reduction of *MMP3* mRNA (Figure 7c) and apparent decrease of MMP3 protein (Figure 7d). Collectively, our results demonstrate that miR-145 targets *MMP3*, as well as *Oct4*, *Lin28*, *Nanog*, *Sox2* and *ZEB1* which are upregulated by linc-DYNC2H1-4 in pancreatic cancer cells. To determine whether linc-DYNC2H1-4 exerted its function through miR-145 in pancreatic cancer cells, rescue experiments were conducted. Again, linc-DYNC2H1-4 transfection increased the protein levels of MMP3, Oct4, Lin28, Nanog, Sox2, ZEB1 and vimentin, and decreased the protein level of E-cadherin in BxPC-3-Gem cells. When the cells were co-transfected with miR-145, all the molecular alterations were rescued to comparable level with control groups (Figure 7e). Similar effects were confirmed in PANC-1 cells, showing miR-145 could block the function of linc-DYNC2H1-4 (Figure 7f). Our data strongly suggest that linc-DYNC2H1-4 acts as a sponge of miR-145 to upregulate the expression of its targets, MMP3, Oct4, Lin28, Nanog, Sox2 and ZEB1, thereby promoting EMT progression and CSC formation in pancreatic cancer cells.

## Discussion

Conventional treatment for cancers mainly targets the differentiated tumor cells; however, in a significant number of patients, cancer cells will acquire drug resistance after standard therapies, resulting in tumor recurrence and metastasis. Mounting evidence has demonstrated that both EMT phenotypic cells and CSCs are associated with the acquisition of these malignant properties.^21, 22, 23, 24, 25^ EMT cells can serve as the source of CSC, and the existence of CSC also confers EMT phenotype.^26^ Overlapping of these two characters suggests that they might be controlled by similar molecules/pathways. For examples, ZEB1 and ZEB2, the key regulators for EMT process, have been proven to maintain stemness properties,^27^ while stem cell maker Lin28 can induce EMT *via* downregulation of let-7.^28^ Our results and the work from others showed that pancreatic cancer cells accumulated EMT and stemness phenotypic cells while developing gemcitabine resistance (Figures 1 and 2).^29, 30^ Therefore, it is important to identify which factor could promote EMT and CSC formation and uncover the mechanistic role of such factor for the development of novel and targeted therapies. This study identified the linc-DYNC2H1-4 as a driver of EMT and CSC formation in pancreatic cancer cells. Linc-DYNC2H1-4 is an intergenic non-coding RNA about 281 nt in length, and has been originally discovered in human liver.^31^ We found that linc-DYNC2H1-4 was differentially expressed in pancreatic cancer cells with different EMT and stemness potentials. Overexpression of linc-DYNC2H1-4 promoted migration and invasion as well as pacreatosphere-forming ability in gemcitabine-sensitive pancreatic cancer cells. Knockdown of linc-DYNC2H1-4 suppressed the acquisition of EMT phenotypes and CSC properties in gemcitabine-resistant cells (Figures 4 and 5).

Emerging evidence demonstrate that lncRNA may serve as miRNA sponge. Recent study has unveiled that the cytoplasmic localization is critical for lncRNA sponge efficacy.^32^ The cytosolic localization of linc-DYNC2H1-4 supported its function as miRNA sponge. Mutation analysis revealed that linc-DYNC2H1-4 binding to miR-145 via specific sequences (Figure 6). Sponge lncRNA might reduce miRNA expression at post transcription level, for example, HSUR 1 directs the degradation of mature miR-27 in a sequence-specific and binding-dependent manner.^33^ Our results showed that mature miR-145 was reduced by linc-DYNC2H1-4. Overexpression of linc-DYNC2H1-4 led to decreased expression of miR-145 while knockdown of linc-DYNC2H1-4 resulted in opposite effects. In addition, BxPC-3-Gem and PANC-1 with high level of linc-DYNC2H1-4 showed low level of miR-145 compared with BxPC-3 and MIA PaCa-2 cells (Figure 6). Our results clearly demonstrate that linc-DYNC2H1-4 competes with miR-145 and reduces its mature level, but the mechanism that can explain miR-145 reduction by linc-DYNC2H1-4 is unclear in this study.

miR-145 is established as a tumor suppressor,^34, 35^ targeting embryonic transcription factors including Lin28, Nanog, Sox2 and Oct4^17, 18, 19^ in various cancer models. In addition, miR-145 also inhibits the EMT key regulator, ZEB1 expression.^20^ We verified that miR-145 targeted these genes that are important for EMT and CSC formation in pancreatic cancer (Figure 7). As a sponge of miR-145, linc-DYNC2H1-4 should be able to relieve the expression of miR-145 targets. Ectopic expression of linc-DYNC2H1-4 in parental BxPC-3 cells with high miR-145 expression significantly elevated the Lin28, Nanog, Sox2, Oct4 and ZEB1 expressions while knockdown of linc-DYNC2H1-4 in BxPC-3-Gem cells with low miR-145 expression showed the opposite effects (Figures 4 and 5). Furthermore, upregulation of these miR-145 targets by linc-DYNC2H1-4 was reverted by miR-145 overexpression, demonstrating that linc-DYNC2H1-4 can compete with miR-145 to release its targets that are associated with EMT and CSC properties (Figures 7e and f). As a sponge, lncRNAs is often reported to relieve one specific target of miRNA, for example, Hotair, and H19 have been reported to compete with miR-331-3p and miR-138/miR200a to regulate expressions of Her2 and vimentin/ZEB, respectively.^36, 37^ However, integrative analysis revealed that sponge regulation by lncRNA had a widespread influence on the expression of protein-coding cancer driver genes, which was not a simple one-to-one.^32^ LncRNA might sponge several miRNAs or single miRNA that targets multiple protein-coding genes, thus revert multiple genes expressions. Our data showed that linc-DYNC2H1-4 could relieve multiple genes that are important in EMT and CSC regulation by sponge miR-145.

MMP3 has been shown to stimulate EMT process *in vitro* and in transgenic mice.^38, 39, 40^ Exposure to MMP3 leads to upregulation of Snail, a key regulator of EMT process.^40^ As a nearby gene of linc-DYNC2H1-4, MMP3 expressed differentially in accordance with linc-DYNC2H1-4 levels in gemcitabine-sensitive and resistant cell lines. Mechanically, we found that miR-145 was also involved in the regulation of linc-DYNC2H1-4 towards MMP3. MiR-145 binding sites were detected in the 3′ UTR of *MMP3* gene. Overexpression of miR-145 decreased MMP3 expression in gemcitabine-resistant cell line (Figures 7c and d). Moreover, MMP3 upregulation induced by linc-DYNC2H1-4 was downregulated by miR-145, demonstrating that competing with miR-145 is one of the mechanisms for linc-DYNC2H1-4 to regulate *MMP3*.

In summary, our results demonstrate that linc-DYNC2H1-4 is involved in the regulation of both EMT and stemness in pancreatic cancer cells. It upregulates the nearby gene *MMP3* and EMT regulator *ZEB1* as well as embryo factor *Lin28*, *Oct4*, *Nanog* and *Sox2*, thus promotes EMT and CSC properties. Mechanically, it could compete with miR-145 that targets *MMP3*, *ZEB1*, *Lin28*, *Nanog*, *Sox2*, *Oct4* to restore these EMT and CSC-associated genes expressions.

## Materials and methods

### Cell lines and cell culture

Pancreatic cancer cell lines BxPC-3, AsPC-1 and PANC-1 were purchased from the Chinese Type Culture Collection, Chinese Academy of Sciences (Shanghai, China), and cultured in RPMI 1640 medium (Gibco, BRL Co. Ltd., USA) with 10% fetal bovine serum (Gibco, Grand Island, NY, USA) in a humid atmosphere containing 5% CO_2_ at 37 °C. The drug-resistant pancreatic cancer cell line BxPC-3-Gem was obtained by treating parental BxPC-3 cells with increasing dosages of gemcitabine (LC Laboratories Company, Woburn, USA) intermittently for 16 months, and then persistently cultured in medium containing 50 nmol/l of gemcitabine.

### Constructs and transfection

Linc-DYNC2H1-4 overexpression vector (p-linc-DYNC2H1-4) was constructed by cloning a PCR fragment of 281 bp into the pcDNA3.1(+) vector.

Dual luciferase reporter construct containing linc-DYNC2H1-4 with two predicted binding sites to miR-145 (psiCHECK2-WT) was generated by cloning the 281 bp PCR fragment into psiCHECK2. Each binding site was mutated using Fast Site-Directed Mutagenesis Kit (TIANGEN, Beijing, China) to generate mutant constructs (psiCHECK2-Mut1, psiCHECK2-Mut2). Construct with mutations at both binding sites (psiCHECK2-Mut1+2) was generated by PCR using psiCHECK2-Mut1 as the template and Mut2 primers. Construct psiCHECK2-MMP3 3′ UTR was generated by cloning a 324 bp fragment of MMP3 3′ UTR into the psiCHECK2. The siRNAs: si-linc-DYNC2H1-4 and negative control si-NC were synthesized by GenePharma (Shanghai, China). The primer and siRNA sequences were listed in Supplementary Table 1.

Transient transfection was performed by using a standard protocol from the Lipofectamine 3000 (Invitrogen, Eugene, OR, USA).

### Dual luciferase reporter assay

BxPC-3-Gem cells were seeded in triplicate in 24-well plate and transfected with the above luciferase reporter constructs together with p-miR-145 or empty vector pacAd5 miR-GFP-Puro (Generous gifts of Dr. Weiming Tian, Harbin Institute of Technology, China) for 24 h. Renilla luciferase activity was normalized to the firefly luciferase activity by using the Dual Luciferase Reporter Assay System (Promega, Madison, WI, USA).

### MTT assay

After transfection, cells were planted in 96-well plates, and incubated with various concentrations of gemcitabine for 72 h. MTT (0.5 mg/ml) was added to each well and incubated for 4 h, followed by colorimetric analysis (wavelength, 490 nm) on a microplate spectrophotometer (Bio-Rad, Hercules, CA, USA).

### Sphere formation assay

Cells (500/well) were seeded into 6-well ultra-low attachment plates (Corning, Inc., Corning, NY, USA), and cultured in suspension in DMEM/F12 (Gibco, Grand Island, NY, USA) supplemented with 2% B27, 10 ng/ml EGF and 10 ng/ml basic FGF (Gibco, MD, USA). After 7 days of culture, primary pancreatospheres were grown for 7 days followed by centrifugation and digestion with StemPro Accutase Cell Dissociation Reagent (Invitrogen, San Diego, CA, USA) and then reseeded (500 cells/well) to develop secondary spheres after another 7 days of growth. Same procedure was repeated for tertiary pancreactospheres. Spheres with diameter >75 μm were counted.

### Colony formation assay

Cells (500, 250 or 125/well) were seeded into 6-well plates and cultured for 14 days without disturbance. Colonies were fixed in formaldehyde and stained with crystal violet. Colonies with over 50 cells were counted.

### Preparation of nuclear and cytoplasmic fractions

The nuclear and cytoplasmic fractions were prepared as described previously.^41^ Briefly, the cells were centrifuged at 1000 ×  *g* for 5 min after washing twice with cold phosphate-buffered saline (PBS). The pellet was re-suspended in prechilled cell disruption buffer (1.5 mM MgCl_2_, 10 mM KCl, 20 mM Tris-Cl, 1 mM DTT) and incubated on ice for 10 min, followed by homogenization to disrupt the cell membranes. The homogenate was visually inspected under a microscope to ensure that ⩾90% of the cells had broken cellular membranes while very few broken nuclei, then the homogenate was added with 0.1% Triton X-100. The cell nuclei were separated from the cytosol by centrifuging the homogenate at 1500 ×  *g* for 5 min. RNAs from both fractions were extracted using TRIzol reagent (Invitrogen, Eugene, OR, USA).

### Quantitative real-time PCR

Total RNA was prepared from cells or tissue specimens using TRIzol reagent and treated with gDNA eraser (Gibco BRL, Grand Island, NY, USA) to remove the genomic DNA according to the manufacturer's protocol. For quantification of miR-145 expression, microRNAs were extracted using miRNA Isolation Kit (Ambion Inc, Foster, CA, USA) and polyadenylated by using Poly (A) Tailing Kit (Ambion, Waltham, MA, USA). RNA was reverse transcribed to cDNA using Reverse Transcription Kit (Takara, Otsu, Shiga, Japan). RT-qPCR was performed using SYBR Premix Ex Taq (Takara, Otsu, Shiga, Japan) on ViiA7 Real-time PCR System (Applied Biosystems Inc., Foster City, CA, USA). GAPDH was used as internal control for lncRNA and mRNA, while U6 RNA was used as endogenous control for miRNA. Each sample was analyzed in triplicate. The primers are listed in Supplementary Table 2.

### Western blotting assay

Cell lysis and western blot were conducted as described previously.^42^ Briefly, 40–100 μg proteins per well were resolved by SDS-PAGE and transferred on PVDF (Millipore, Darmstadt, Germany) membranes. After 1 h of blocking, membranes were incubated with the following primary antibodies: mouse anti-GAPDH (KC-5G4) (KANGCHEN, Shanghai, China), *β*-actin (Santa Cruz, CA, USA), and MMP3, Oct4, Sox2, Nanog, E-cadherin, vimentin, ZEB1, Lin28 (Proteintech, Wuhan, China) at 4 °C over night. After washing, membranes were incubated with secondary antibodies (Cell Signaling Technology, USA). The signals were detected using ECL (APPLYGEN, Beijing, China) and visualized using LI-COR (Biosciences, Lincoln, NE, USA).

### Transwell assay

Cells (2.5 × 10^4^/well) suspended in serum-free medium were seeded in the upper chamber of Transwell (Costar Corp., Cambridge, MA, USA), and allowed to translocate toward medium containing 20% FBS in the lower chamber for 48 h. The cells that migrated to the lower surface were fixed with 4% formaldehyde and stained with 0.5% crystal violet. Cells were counted in five photographed fields. In the invasion assay, filters were pre-coated with Matrigel (BD Biosciences, Franklin Lakes, NY, USA). Both cell migration and invasion assays were performed in triplicate and repeated three times.

### LncRNA microarray and data analysis

LncRNA/mRNA expression microarray analysis for BxPC-3 and BxPC-3-Gem cell lines was performed using Agilent Array platform (Kangchen Bio-tech, Shanghai, China). Agilent Feature Extraction software (version 11.0.1.1) was used to analyze the acquired array images. Quantile normalization and subsequent data processing were performed using the GeneSpring GX v11.5.1 software package (Agilent Technologies, Santa Clara, CA, USA). As the results of lincRNA and their nearby genes array analysis, values presented are log2 fold change. We searched features of the nearby genes, and categorized as CSC and EMT.

### Patients and specimens

Frozen PDAC samples and adjacent noncancerous tissues were collected from 18 patients diagnosed with PDAC at the Affiliated Tumor Hospital of Harbin Medical University (Harbin, China). All patients provided written informed consent and ethical consent was granted from the Committees for Ethical Review of Research involving Human Subjects of Harbin Medical University.

### *In vivo* tumorigenicity

The animal experimental procedures were conducted strictly in accordance with the Guide for the Care and Use of Laboratory Animals, and approved by the Animal Care and Use Committee of the Harbin Institute of Technology. Male athymic BALB/c nude mice (4–5 weeks old) were obtained from Cancer Institute of the Chinese Academy of Medical Science (Beijing, China). BxPC-3 or BxPC-3-Gem cells at different numbers (10^3^, 10^5^ and 10^7^ per inoculation) were subcutaneously implanted in the symmetrical posterior dorsal flank region of nude mice (*n*=4). The mice were sacrificed 3 weeks after injection, tumor weight was measured. In another experiment, PANC-1 cells (10^6^ per inoculation, *n*=4) and BxPC-3 cells (10^6^ and 10^7^ per inoculation, *n*=4) were inoculated in nude mice. The mice were sacrificed 8 weeks after injection.

### Statistical analysis

SPSS 21.0 statistical software was used for data statistical analysis. Results were described as mean±S.D. Statistical significance between two groups was determined with Student's *t*-test. The level of significance was set at *P*<0.05.

## Figures and Tables

**Table 1:** Tumorigenicity of PANC-1 and BxPC-3 cells in BALB/c nude mice

**Cell line**	**Cells/mice**	**Tumor volume (mm**^**3**^)	**Tumor weight (mg)**	**Incidence**
PANC-1	1 × 10^6^	314.3.0±131.8	345.0±41.9	4/4
BxPC-3	1 × 10^6^	0	0	0/4
BxPC-3	1 × 10^7^	218.2±51.5	226.9±25.6	4/4
