# Author Correction: Linc-DYNC2H1-4 promotes EMT and CSC phenotypes by acting as a sponge of miR-145 in pancreatic cancer cells

**DOI:** 10.1038/s41419-019-1852-2

**Published:** 2019-08-13

**Authors:** Yuran Gao, Zhicheng Zhang, Kai Li, Liying Gong, Qingzhu Yang, Xuemei Huang, Chengcheng Hong, Mingfeng Ding, Huanjie Yang

**Affiliations:** 1 School of Life Science and Technology, Harbin Institute of Technology, Harbin, China; 2 Department of General Surgery, Fourth Affiliated Hospital of Harbin Medical University, Harbin, China

**Correction to:** **Cell Death & Disease**

10.1038/cddis.2017.311

published online 13 July 2017

After publication of this article, the below errors were noticed:

- The SOX2 primer is incorrect in Table S2.

**Table S2:** Primers for qRT-PCR

Targets	Sequences	
Oct4	F: 5′-CGCCGTATGAGTTCTGTG-3′	R: 5′-GGTGATCCTCTTCTGCTTC-3′
Lin28	F: 5′-AAAGGAGACAGGTGCTAC-3′	R: 5′-ATATGGCTGATGCTCTGG-3′
Nanog	F: 5′-AAGAACTCTCCAACATCCTGAAC-3′	R: 5′-CCTTCTGCGTCACACCATT-3′
Sox2	F: 5′-AGTTGGACAGGGAGATGGC-3′	R: 5′-AACCTTCCTTGCTTCCACG-3′
MMP3	F: 5′-TTCCGCCTGTCTCAAGATGATAT-3′	R: 5′-AAAGGACAAAGCAGGATCACAGTT-3′
MMP1	F: 5′-AAATGCAGGAATTCTTTGGG-3′	R: 5′-ATGGTCCACATCTGCTCTTG-3′
MMP27	F: 5′-GTTTAGAAGTGTGGAGCAAAGTCACT-3′	R: 5′-ATAGCGAGGACACCGACCAT-3′
ZEB1	F: 5′-GTTACCAGGGAGGAGCAGTGAAA-3′	R: 5′-GACAGCAGTGTCTTGTTGTTGTAGAAA-3′
Vimentin	F: 5′-AGTCCACTGAGTACCGGAGAC-3′	R: 5′-CATTTCACGCATCTGGCGTTC-3′
E-cadherin	F: 5′-TACACTGCCCAGGAGCCAGA-3′	R: 5′-TAATCCGGACACTGGTGCCA-3′
Linc-DYNC2H1-4	F: 5′-GCACGGCAGGAAGCAAG-3′	R: 5′-CCAGCCTCCTCTGCCTTGT-3′
DYNC2H1-4	F: 5′-GTGCTGAAAGGGATCGTGTG-3′	R: 5′-GGCTTTTGTTGTCATTAGAT-3′
GAPDH	F: 5'-TGCACCACCAACTGCTTAGC-3'	R: 5'-GGCATGGACTGTGGTCATGAG-3'
U6	F: 5′-CGCTTCGGCAGCACATATACTA-3′	R: 5′- CGCTTCACGAATTTGCGTGTCA-3′
MT-RNR1	F: 5′-CCTCCCCAATAAAGCTAAAA-3′	R: 5′- GCTATTGTGTGTTCAGATAT-3′
miR-145	Poly(T) adaptor: GCTGTCAACGATACGCTACCTAACGGCATGACAGTGTTTTTTTTTTTTTTTVN^a^	
	F: 5′-GTCCAGTTTTCCCAGGAATCCCT-3′	R: 5′- GCTGTCAACGATACGCTACCTA-3′

^a^ V=G, C, A; N=G, A, C, T
- The Poly(T) adaptor sequence of reverse transcription for miR-145 detection is missing in Table S2.

The corrected table is provided below. This error did not impact the conclusions of the article. We apologize for any confusion or inconvenience to the readers.

