# Supplementary material for: Linc-DYNC2H1-4 promotes EMT and CSC phenotypes by acting as a sponge of miR-145 in pancreatic cancer cells
Source: Cell Death Dis. 2017 Jul 13;8(7):e2924–. doi: 10.1038/cddis.2017.311 (PMC5550858; doi:10.1038/cddis.2017.311)
Supplement: Supplementary Tables [file cddis2017311x1.docx]

**Table S1. Primers for vector construction and siRNAs**

| **Vector/siRNAs** | **Sequences** | |
| --- | --- | --- |
|  | **Forward** | **Reverse** |
| p-linc-DYNC2H1-4 | 5′-CGCGGATCCGGGCAGCCCTGAA  GCAGGCAA-3′ | 5′-CCGGAATTCCCTGTTTGTAAGAT  AATTCC-3′ |
| psiCHECK2-WT | 5′-CGACTCGAGGGGCAGCCCTGAA  GCAGGCAA-3′ | 5′- CGAGCGGCCGCCCTGTTTGTAA  GATAATTCC-3′ |
| psiCHECK2-Mut1 | 5′-TGAAGCAGGCAAAGGAACGGAT  CCACGGCAGGAAGCAAGT-3′ | 5′-ACTTGCTTCCTGCCGTGGATCCG  TTCCTTTGCCTGCTTCA-3′ |
| psiCHECK2-Mut2 | 5′-GGAGAGCCCAAGGATGAATCGA  TAGAAGTAATTAATGGCA-3′ | 5′-TGCCATTAATTACTTCTATCGATT  CATCCTTGGGCTCTCC-3′ |
| psiCHECK2-MMP3 3′UTR | 5′- TCGAGTTTAAACGAGATATGTAG  AAGGCACAATAT-3′ | 5′-TCGAGCGGCCGCATAAAATAACT  GACAAATCGTCT-3′ |
| si-linc-DYNC2H1-4 | 5′- UCCUGAAGCCUAAAGGGAATT-3′ |  |
| si-NC | 5′- UUCUCCGAACGUGUCACGUTT-3′ |  |

**Table S2. Primers for qRT-PCR**

| **Targets** | **Sequences** | |
| --- | --- | --- |
|  | **Forward** | **Reverse** |
| Oct4 | 5′-CGCCGTATGAGTTCTGTG-3′ | 5′-GGTGATCCTCTTCTGCTTC-3′ |
| Lin28 | 5′-AAAGGAGACAGGTGCTAC-3′ | 5′-ATATGGCTGATGCTCTGG-3′ |
| Nanog | 5′-AAGAACTCTCCAACATCCTGAAC-3′ | 5′-CCTTCTGCGTCACACCATT-3′ |
| Sox2 | 5′-AGTTGGACAGGGAGATGGC-3′ | 5′-AACCTTCCTTGCTTCCACG-3′ |
| MMP3 | 5′-TTCCGCCTGTCTCAAGATGATAT-3′ | 5′-AAAGGACAAAGCAGGATCACAGTT-3′ |
| MMP1 | 5′-AAATGCAGGAATTCTTTGGG-3′ | 5′-ATGGTCCACATCTGCTCTTG-3′ |
| MMP27 | 5′-GTTTAGAAGTGTGGAGCAAAGTCACT-3′ | 5′-ATAGCGAGGACACCGACCAT-3′ |
| ZEB1 | 5′-GTTACCAGGGAGGAGCAGTGAAA-3′ | 5′-GACAGCAGTGTCTTGTTGTTGTAGAAA-3′ |
| Vimentin | 5′-AGTCCACTGAGTACCGGAGAC-3′ | 5′-CATTTCACGCATCTGGCGTTC-3′ |
| E-cadherin | 5′-TACACTGCCCAGGAGCCAGA-3′ | 5′-TAATCCGGACACTGGTGCCA -3′ |
| Linc-DYNC2H1-4 | 5′-GCACGGCAGGAAGCAAG-3′ | 5′-CCAGCCTCCTCTGCCTTGT-3′ |
| DYNC2H1-4 | 5′-GTGCTGAAAGGGATCGTGTG-3′ | 5′-GGCTTTTGTTGTCATTAGAT-3′ |
| GAPDH | 5'-TGCACCACCAACTGCTTAGC-3' | 5'-GGCATGGACTGTGGTCATGAG-3' |
| miR-145 | 5′-GTCCAGTTTTCCCAGGAATCCCT -3′ | 5′- GCTGTCAACGATACGCTACCTA -3′ |
| U6 | 5′-CGCTTCGGCAGCACATATACTA-3′ | 5′- CGCTTCACGAATTTGCGTGTCA -3′ |
| MT-RNR1 | 5′-CCTCCCCAATAAAGCTAAAA-3′ | 5′- GCTATTGTGTGTTCAGATAT-3′ |
| U99 | 5′-CCTCCTTTTCTTGGCGGGGA-3′ | 5′- CGTTTGAGGATAGAACCAGC-3′ |
