# Supplementary material for: Linc-DYNC2H1-4 promotes EMT and CSC phenotypes by acting as a sponge of miR-145 in pancreatic cancer cells
Source: Cell Death Dis. 2017 Jul 13;8(7):e2924–. doi: 10.1038/cddis.2017.311 (PMC5550858; doi:10.1038/cddis.2017.311)
Supplement: Supplementary Figure legend [file cddis2017311x3.doc]

**Figure S1** Sensitivity of pancreatic cancer cells to gemcitabine. MIA PaCa-2 and PANC-1were treated with gemcitabine for 72 h followed by MTT assay.
